# Hydrolytic Unzipping of Boron Nitride Nanotubes in Nitric Acid

**DOI:** 10.1186/s11671-017-1877-3

**Published:** 2017-02-07

**Authors:** Dukeun Kim, Hiroyuki Muramatsu, Yoong Ahm Kim

**Affiliations:** 1 Alan G. MacDiarmid Energy Research Institute, School of Polymer Science and Engineering, Chonnam National University, 77 Yongbong-ro, Gwangju, Buk-gu 61186 Republic of Korea; 2 Water Environment and Civil Engineering, Faculty of Engineering, Shinshu University, Wakasato, 4-17-1, Nagano, Japan

**Keywords:** Boron nitride nanoribbons, Hydrolysis, Nitric acid, Methylene blue

## Abstract

Boron nitride nanoribbons (BNNRs) have very attractive electrical and optical properties due to their unique edge states and width-related properties. Herein, for the first time, BNNRs were produced by a simple reflux of boron nitride nanotubes (BNNTs) in nitric acid containing water, which had led to unzipped sidewalls through hydrolysis. Their high reactivity that originated from edges was verified via a strong interaction with methylene blue.

## Background

Boron nitride nanoribbons (BNNRs) which are strips of the BN nanosheets (BNNSs) with nanoscale widths, possess interesting electrical and optical properties due to their unique edge states and width-related properties. Therefore, a comparison between two dimensional BNNSs with one dimensional tubular BN nanotubes (BNNTs) would be an interesting and significant study [1]. In fact, the BNNRs have adjustable half-metallic property because their bandgaps can be altered by various factors, such as nanoribbon’s width, electric field, and edge functionalization with hydrogen, fluorine, and oxygen atoms [2–6]. BNNSs and BNNTs are electrically insulating, and they have large bandgaps (e.g., 5.5 eV) [7, 8]. BNNRs exhibit higher chemical reactivity [9] than those of BNNSs and BNNTs due to their numerous unsaturated edge atoms, which makes BNNRs a potential candidate in the fabrication of gas sensor. Moreover, BNNRs can be used as fillers in enhancing mechanical property of composites due to strong interfacial interaction between BNNR edges and matrix. Previous study showed that BNNRs exhibited higher electrical conductivity than BNNTs [10]. Accordingly, the BNNRs are highly promising in the fabrication of nanoscale electronic and optoelectronic materials, sensor, catalysts, and functional composites [11–15]. Several procedures for producing BNNRs have been developed via the longitudinal cutting of BNNTs using plasma etching with alkali metal intercalation [10], expansion of intercalated potassium [16], and unzipping of BNNTs during the nanotube synthesis under extreme conditions [11].

The BNNRs were arduously produced by these methods which have certain disadvantages, such as the presence of complicate pre-production step, the necessity of special equipment, and skill. For example, in case of plasma etching method with alkali metal intercalation [10], the BNNTs should be embedded into polymer matrix on a Si substrate. For expanding intercalated potassium method, high temperature furnace above 1500 °C is required [16]. Several researchers including our group have studied simple ways of producing BNNRs without using pre-production step, special treatment, and equipment. Our group discovered that longitudinally unzipped BNNTs can be produced via alcoholysis using alcohol with long alkyl chains under sonication because the chemical interaction between boron atom of BNNTs and oxygen atoms of alcohol lead to generation of alkyl chain-connected BNNRs [17]. However, alkyl chains attached on BNNR edges largely deteriorate their chemical reactivity. Thus, a more desirable method of producing chemically active BNNRs should be developed. Our prior work suggested that BNNTs can be unzipped to BNNRs in an oxygen-rich solution under suitable power.

In the current study, we designed a simple way of producing BNNRs via hydrolysis of BNNTs without using any special equipment. Chemical peeling of BNNTs was achieved by refluxing them in nitric acid. More specifically, a 24-h reflux led to formation of the BNNRs of a considerable amount. The formation of BNNRs was additionally demonstrated by measuring reactivity with methylene blue (MB), which is well known for color pollutant caused by various industries such as textile, leather, paper, and printing [18]. Due to the difference of structures between nanoribbons and nanotubes, the synthesized BNNRs showed higher reactivity toward MB than BNNTs.

## Methods

**Fig. 1:**
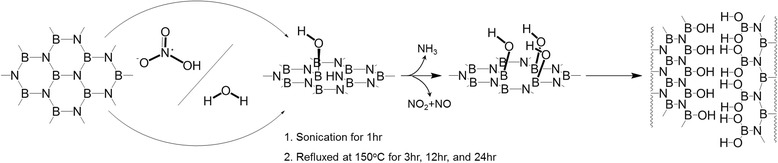
The suggested schematic process for the hydrolysis-assisted unzipping of boron nitride nanotube in nitric acid

The unzipping method presented is based on the hydrolysis of B–N bond (Fig. 1). At first, multi-walled BNNTs (BNNT Inc.) were purified through acid and heating treatments to remove certain impurities, such as catalyst and h-BN powders. The purified sample (5 mg) in 68% HNO_3_ (HNO_3_∙H_2_O, 50 ml, TCI Inc.) solution was sonicated for 90 min using a bath sonicator (Branson 3210, Branson Ultrasonics Corp.) in order to break large clusters of BNNTs into smaller pieces. The solutions were first refluxed at 150 °C for 3, 12, and 24 h followed by vacuum filtration using 1-μm membrane filter (pore size 1.0 μm, diameter 25 mm, Advantec Inc.), and the precipitate was then washed with deionized water several times and finally dried at 50 °C in vacuum oven overnight.

## Results and Discussion

The hydrolysis-assisted unzipping procedure of BNNTs has been schematically described in Fig. 1. Our previous work reported unzipping of BNNTs into BNNRs in alcohol under sonication which indicated that alcohol molecules activated BN bonds through coordination for subsequent hydrolysis [17]. Furthermore Liao et al. [19] showed that chemical sharpening, shortening, and unzipping of BNNTs occurred in water-ammonia mixture because ammonia molecules coordinated to the B atoms as Lewis base, followed by hydrolysis of BN bonds. It was expected that the boron atom in B–N bonds near defect sites were prone to attack by O atoms from unstable (H_3_O)^+^ molecules coming from H_2_O and HNO_3_ at 150 °C, thereby leading to hydrolysis of adjacent borazin units. These phenomena would propagate into the BNNT edge, resulting in the formation of BNNRs. Field emission scanning electron microscopy (FE-SEM, S-4700, Hitachi Ltd., Japan) and field emission transmission electron microscopy (FE-TEM, JEM-2100F, JEOL Ltd., Japan) were used to see the morphological change of BNNTs before and after the hydrolysis. To see the chemical bonding state before and after the hydrolysis, Fourier transform infrared spectroscopy (FT-IR, Nicolet iS10 spectrometer using ATR accessory, Thermo scientific Ltd., Japan) was used. Finally, the UV-visible absorption spectra (Optizen 2120 UV, Mecasys, Korea) were obtained to evaluate the interaction between BNNRs and methylene blue (MB). 

**Fig. 2:**
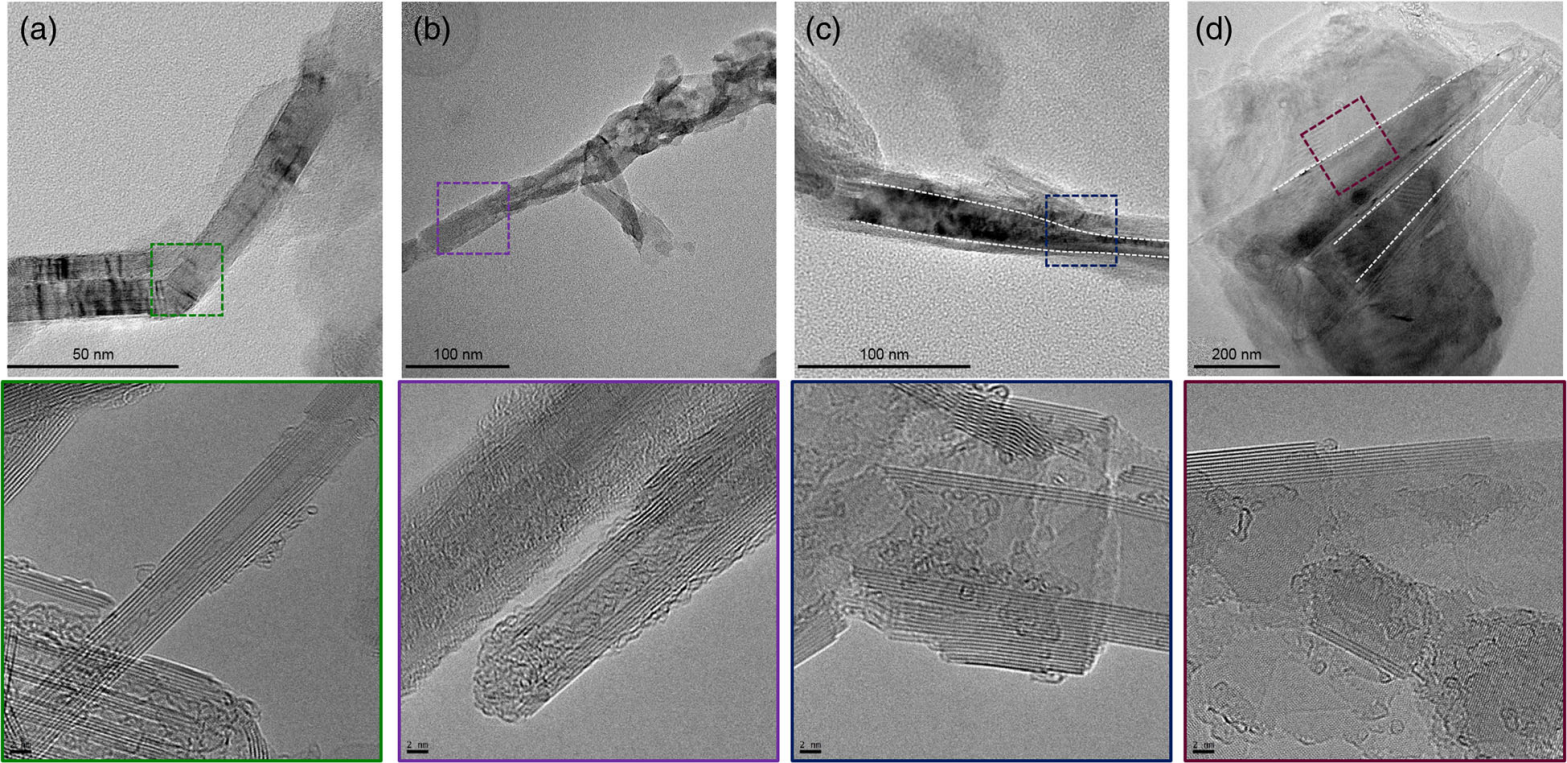
Transmission electron microscopic images of **a** the purified boron nitride nanotubes and samples obtained by refluxing in nitric acid for **b** 3 h, **c** 12 h, and **d** 24 h; enlarged images of **b**, **c**, and **d** indicated partially unzipped BNNTs, respectively

To support our claim the refluxed samples were studied using high-resolution transmission electron microscopy (HR-TEM) in detail (Fig. 2). Even though amorphous-like structure was observed in purified BNNTs (Fig. 2 a), TEM image of BNNTs showed high contrast walls with low contrast inner core and periodic regions (Fig. 2 b), indicating their high quality. However, TEM images of 3-h-refluxed sample (Fig. 2c, d) showed numerous amorphous structures without any darkened region. Thus, it became apparent that chemical peeling of BNNTs occurred via reflux in nitric acid for 3 h. Interestingly, we observed the BNNR-like structure from the 12-h-refluxed sample (see dotted navy square in Fig. 2e). Eventually, narrow BNNRs with large aspect ratio were identified in the 24-h-refluxed sample (Fig. 2g, h). In addition, the yield of BNNRs with regard to BNNTs based on TEM image is roughly 30%. Our detailed TEM observation demonstrated that BNNRs can be produced via chemical unzipping process by refluxing BNNTs in nitric acid.

**Fig. 3:**
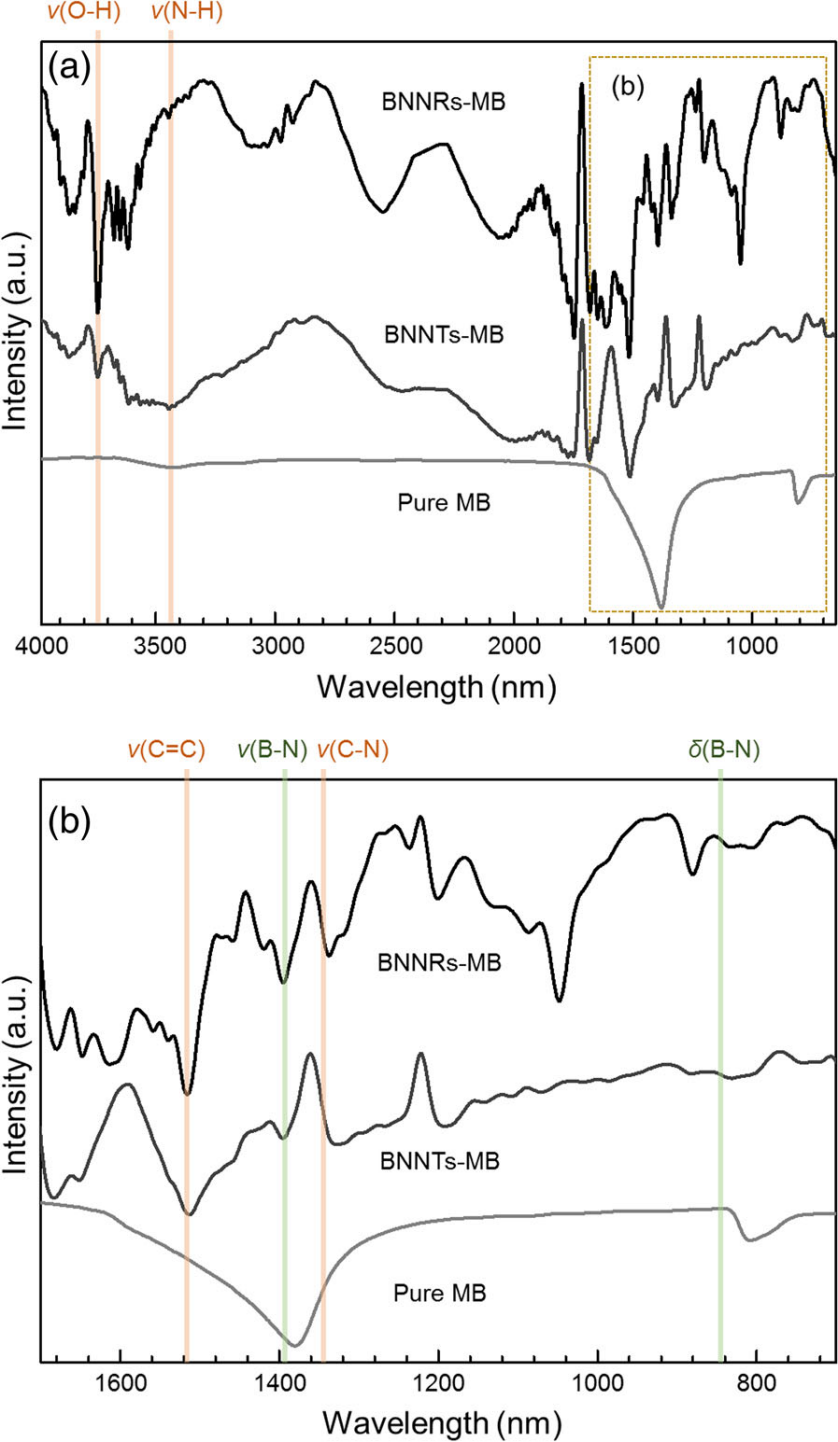
FT-IR spectra of **a** methylene blue, boron nitride nanoribbons, and boron nitride nanoribbons-methylene blue, where the boron nitride nanotubes were produced by refluxing boron nitride nanotubes in nitric acid for 24 h. **b** Their enlarged FT-IR spectra in the range from 1700 to 700 nm

The BNNRs prepared by refluxing BNNTs in nitric acid for 24 h were mixed with MB in ethanol under bath sonication. To obtain information on chemical bonding between BNNRs and MB, the dispersed objects were collected as dry powders and analyzed by FT-IR spectroscopy (Fig. 3). The pure MB exhibited specific absorption peaks at 3444, 1511, and 1328 cm^−1^ corresponding to N–H, C = C, and C–N stretching vibration peaks, respectively. The BNNRs showed the characteristic absorption peaks at 1380 and 808 cm^−1^, which can be assigned to B–N vibration bands parallel (v) and perpendicular (δ) to the major axes [17, 19]. In the BNNRs-MB sample, we observed an upshift of the BN stretching vibration related peak at 1394 cm^−1^. Moreover, the stretching vibration peak coming from N–H from MB was split and upshifted around 3648 cm^−1^, suggesting that nitrogen atom of MB was probably anchored on the BNNRs via electrostatic forces onto negatively charged OH bond on the edges of the BNNRs. Since MB is an ideal planar molecules, π-π interaction [18] between MB (cationic dye, π electron-acceptors) and electron-rich N atoms of the surface of BNNRs is plausible. Such interaction may lead to decrease in intensity of the B–N bending vibration related peak and the upshift in the B–N stretching vibration related peak for the BNNRs-MB sample. The FTIR results verified that MB can be adsorbed onto BNNR edges and/or surfaces via electrostatic attractions and π-π interaction.

**Fig. 4:**
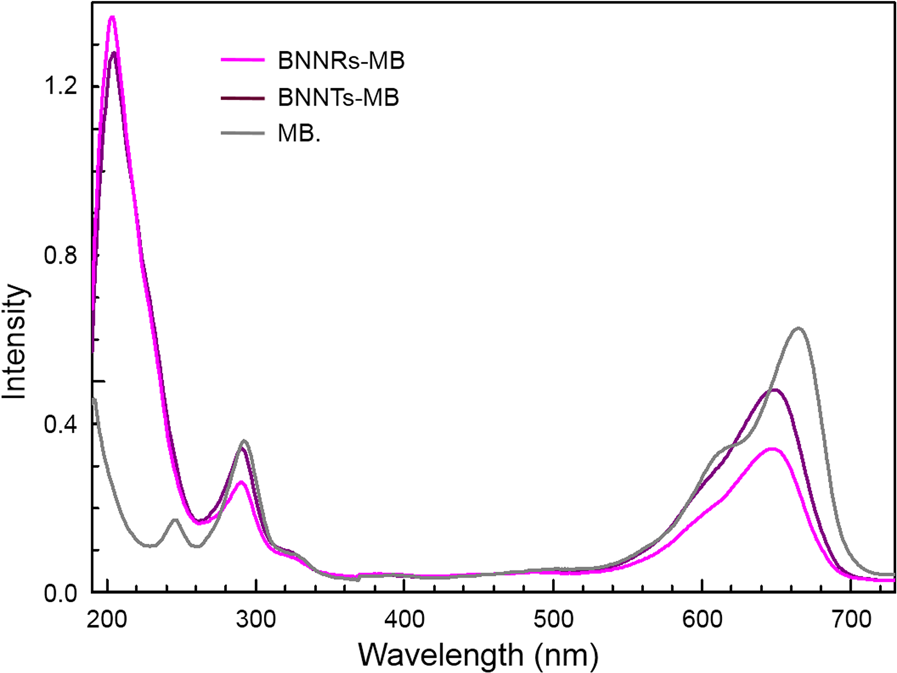
UV–vis absorption spectra of methylene blue, boron nitride nanotubes-methylene blue, and boron nitride nanoribbons-methylene blue in ethanol

The dispersed solutions in ethanol were further investigated using UV-visible spectroscopy to see the interaction between BNNRs and MB (Fig. 4). Pure MB in ethanol showed two strong absorption peaks at 665 and 615 nm which could be associated with MB monomer and dimer in solvent, respectively [20]. However, for BNNTs-MB sample, we observed that both decrease in intensity and downshift in the characteristic MB absorption peak (ca. 648 nm). An absorption peak at 205 nm was also observed which came from dispersed BNNTs. Such kind of change was predominantly observed in BNNRs-MB sample. The absorption peak coming from the MB monomer largely decreased and downshifted to 646 nm. In addition, we observed largely intensified absorption peak coming from BN structure. Such a large change in the absorption peaks for BNNRs-MB sample signifies electron-transfer from MB molecules to BN structure. Thus, the edge sites of BNNRs consisting of OH bonds acted as reaction sites with regard to MB molecules.

## Conclusions

In conclusion, we have demonstrated the ability of producing BNNRs by refluxing BNNTs in 68% nitric acid for 24 h. It was confirmed that oxygen atoms of unstable (H_3_O)^+^, activated by reaction between H_2_O and HNO_3_ at high temperature, were able to bind to electron-deficient boron atoms on the surface, resulting in the hydrolysis of borazin units of BNNTs, and followed by the production of BNNRs. The BNNRs have numerous unique edges which acted as reactive sites with regard to MB via electrostatic attractions and π-π interaction. Even though the yield of BNNRs we produced was quite low, it is expected that our method of producing BNNRs would stimulate many new researchers to study the B-N based nanomaterials as well as their surface chemistry.

## Abbreviations

BNNRs: Boron nitride nanoribbons
BNNSs: BN nanosheets
BNNTs: BN nanotubes
MB: Methylene blue
